# Multifaceted Music Therapy for Depression in Dementia: A Network Meta-Analysis of Randomized Controlled Trials

**DOI:** 10.3390/ejihpe14020024

**Published:** 2024-02-11

**Authors:** Berne Ting, Daniel Tzu-Li Chen, Wei-Ti Hsu, Chia-Lin Tsai, Ikbal Andrian Malau, Sheau-Ling Lee, Li Jingling

**Affiliations:** 1 Ph.D. Program for Aging, College of Medicine, China Medical University, Taichung 40402, Taiwan; 2 Mind-Body Interface Laboratory (MBI-Lab), China Medical University Hospital, Taichung 40402, Taiwan; u105023415@cmu.edu.tw (D.T.-L.C.); ikbalgan@gmail.com (I.A.M.); 3 M.D.-Ph.D. Program, College of Medicine, China Medical University, Taichung 40402, Taiwan; 4 School of Chinese Medicine, College of Chinese Medicine, China Medical University, Taichung 40402, Taiwan; 5 Graduate Institute of Biomedical Sciences, College of Medicine, China Medical University, No. 91, Xue-Shi Road, North District, Taichung 40402, Taiwan; u108305203@cmu.edu.tw (W.-T.H.); u9702602@cmu.edu.tw (C.-L.T.); 6 Department of Anesthesiology, China Medical University Hospital, Taichung 40402, Taiwan; 7 National Health Research Institutes, Miaoli 35053, Taiwan; sllee@nhri.edu.tw

**Keywords:** dementia, depression, randomized controlled trials, music therapy

## Abstract

This study aimed to assess the efficacy of various music therapy interventions in ameliorating depressive symptoms in dementia patients, utilizing a network meta-analysis approach. We rigorously selected randomized controlled trials focused on music therapy for dementia with depressive symptoms from major electronic databases. The primary outcome measured was the impact on depressive symptoms, with the secondary outcome evaluating dropout rates across different intervention groups and standard care control groups. The research protocol has been duly registered with PROSPERO (Registration ID: CRD42023393059). Our network meta-analysis incorporated 14 randomized controlled trials involving a total of 1080 participants and examined a range of interventions, including active music therapy, listening to music, rhythmic music therapy, singing, and tailored music interventions. The analysis revealed that active music therapy combined with singing emerged as the most effective intervention, demonstrating a significant improvement in depressive symptoms in dementia patients (Standardized Mean Difference [SMD] = −0.89, 95% Confidence Interval [CI]: −1.48 to −0.30). In contrast, listening to music alone showed a smaller effect (SMD = −0.26, 95% CI: −0.71 to 0.20). This study was particularly noteworthy for not showing higher dropout rates compared to standard care, indicating its feasibility and acceptability in clinical settings. The findings of our study indicate that active music therapy combined with singing is an effective approach to reducing depressive symptoms in dementia patients, potentially due to enhanced social interaction. These results offer new perspectives for dementia care, suggesting a promising direction for further research and clinical application.

## 1. Introduction

Dementia and depression are highly prevalent and often co-occurring conditions among the elderly, with depression representing the most common health issue impacting patients with dementia [1]. The depression associated with dementia is linked to increased personal distress, cognitive and functional impairments, and elevated mortality rates [2]. In individuals with dementia experiencing depression, the condition’s severity and the patient’s quality of life are significantly affected, thereby heightening the need for care [3,4,5,6,7].

It has been established that music offers multiple benefits for depression in dementia, eliciting a wide range of psychological and physiological responses that enhance physical activities, thereby improving physiological and psychological health outcomes and quality of life [8,9,10,11]. It also aids in improving communication and relationships between dementia patients and their family caregivers, reducing caregiver burden and ameliorating psychological symptoms in family caregivers [8]. However, there is a range of music therapy modalities, such as engaging in music classes, singing, listening to music, and participating in rhythmic activities [12,13,14]. While current meta-analyses suggest that music activities broadly benefit the psychological well-being of individuals with dementia, they fall short of pinpointing the specific music activities or genres that are most effective [15,16,17]. Understanding which musical interventions are most effective for alleviating depression in dementia is key to developing effective rehabilitation programs.

Network Meta-Analysis (NMA) embodies a statistical methodology that concurrently evaluates multiple therapy interventions, thereby facilitating the ascertainment of the most effective treatment approaches [18]. Initially, this method involves the aggregation and systematic categorization of various established music therapy interventions. A network model is then devised to allow for the comparative assessment of these interventions, ranking their efficacies. Direct comparisons are made when studies explicitly compare different interventions against each other. In scenarios lacking direct comparisons between interventions, indirect comparisons are inferred through a common comparator. For example, imagine a relay race where Team A finishes faster than Team B by 15 s, and Team B outpaces Team C by 10 s. Direct comparisons are drawn between the teams’ performances. Indirectly, it can be surmised that Team A is likely faster than Team C by around 25 s, despite the absence of a direct race between them. NMA scrutinizes for significant statistical differences between comparisons that have both direct and indirect evidence, ensuring internal consistency [19,20]. By analyzing studies within a designated time frame, NMA can forecast which music therapy interventions may elicit statistically significant improvements in depressive symptoms in dementia over a specified duration. The goal of this study, achieved through our Network Meta-Analysis (NMA), is to establish a clear effectiveness hierarchy among various music therapy interventions for alleviating depression in dementia. Additionally, it aims to estimate the time frame required to observe statistically significant changes. These critical insights are vital in identifying the most appropriate music therapy interventions for effectively reducing depressive symptoms in dementia patients.

## 2. Materials and Methods

This study was meticulously conducted in accordance with the Preferred Reporting Items for Systematic Reviews and Meta-Analyses extensions for Network Meta-Analysis (PRISMA NMA) guidelines [21]. The protocol was registered with PROSPERO, the International Prospective Register of Systematic Reviews (Registration ID: CRD42023393059).

### 2.1. Database Searches and Study Identification

For the identification of relevant studies, a comprehensive search was conducted in four electronic databases: PubMed, Embase, Web of Science, and the Cochrane Library. The time frame for the search spanned from the inception of each database to October 2023. The Boolean search terms applied were music AND depression AND dementia OR Alzheimer’s disease. The search was designed to include all studies that addressed depression in dementia and involved music therapy (MT) interventions.

Initial screening was conducted to remove duplicates and filter out articles not centrally focused on depression in dementia. Following this, a manual search was conducted, and the reference lists of various review articles [17,22,23,24,25,26,27,28] were examined for additional relevant studies. The titles and abstracts of the filtered articles were then assessed for relevance by two independent reviewers (Ting and Hsu). In cases of non-agreement between reviewers, a third party (Li) was called upon to achieve consensus and complete the selection process. This stepwise process ensures that all studies included in the review are pertinent and meet the established eligibility criteria.

### 2.2. Inclusion and Exclusion Criteria

This NMA was guided by the PICO model (Population, Intervention, Comparison, Outcome), encompassing the following criteria: P—patients with depression in dementia; I—music therapy; C—any control group or alternative non-pharmacological intervention; O—standard measures of depression in dementia. Articles included in the analysis were required to meet the following criteria: (1) Randomized Controlled Trials (RCTs); (2) The intervention group received music therapy incorporating three elements of rhythm, melody, and harmony—while the control group received standard care, no treatment, or non-musical interventions; (3) The outcome assessments included measures of depression; and (4) Participants were diagnosed with dementia. Articles not meeting these criteria were excluded: (1) Publications such as review articles, medical protocols, conference papers, case reports, letters, editorials, pilot studies, and initial findings from ongoing research; (2) Studies where music therapy was combined with other therapies or used as a part of complementary or alternative treatments; (3) Control groups that included any accepted element of music; and (4) Studies lacking a primary outcome analysis. Finally, full texts of the eligible articles were used for the final network meta-analysis.

### 2.3. Modeling for Network Meta-Analysis

In this NMA, the model construction was guided by specific principles. To prevent excessive heterogeneity, we limited pairwise comparisons to music versus music or music versus standard care. Comparisons between music and various invasive treatments (e.g., electrotherapy, laser light injections, etc.) or nutritional supplements were excluded. Including additional treatments could lead to different network geometries due to the diversity of considered interventions, potentially resulting in inconsistent outcomes in the NMA [29]. In our study, the categorization of music types was based on discussions between two authors (Ting and Hsu) about the actual music prescription content. Any disagreements in categorization were resolved through discussions with a third author (Li) to reach a consensus.

### 2.4. Methodological Quality Appraisal

In assessing the methodological integrity of the included studies, we employed the Cochrane Collaboration’s Risk of Bias Tool for Randomized Trials (RoB 2, version 2, London, UK) [30]. This instrument scrutinizes various pivotal aspects of research quality, such as the randomization process, adherence to intervention procedures, management of missing outcome data, the precision of outcome measurement, the likelihood of selective reporting, and the general risk of bias in the study.

### 2.5. Primary Outcome: Improvement in Depression among Dementia Patients

The primary outcome of this study was the improvement in depression symptoms in individuals with dementia, quantified using the standardized mean difference. The preferred scales for measurement were the Geriatric Depression Scale (GDS) [31] and the Cornell Scale for Depression in Dementia (CSDD) [32], followed by the Montgomery–Åsberg Depression Rating Scale (MADRS) [33]. The Beck Depression Inventory (BDI) [34] and the Neuropsychiatric Inventory (NPI) [35] were also utilized, albeit as secondary options. This hierarchical approach to the choice of scales was adopted to ensure consistency and accuracy in the assessment of depression severity across the study population.

### 2.6. Secondary Outcome: Risk Difference in Dropout Rates

The secondary outcome measure was the risk difference in dropout rates among participants in music therapy, providing a clear indicator of participant retention. For example, if individuals enrolled in a specific music therapy program aimed at improving depression symptoms in dementia experience a 12% dropout rate, whereas the control group receiving standard care has a 7% dropout rate (which might lead some to seek musical activities independently), the risk difference in dropout rates would be 5% [36]. This difference is important for evaluating the engagement and feasibility of music therapy interventions for dementia with depression.

### 2.7. Data Extraction, Management, and Conversion

Two researchers (Ting and Hsu) independently extracted data, which included participant demographics, study designs, music therapy details, and study outcomes. If needed data were missing in published studies, we tried to obtain these data directly from the authors. Our data handling methods followed the Cochrane Handbook’s guidelines and advice from medical research [19,37,38,39,40]. This process made sure our data were consistently and carefully managed, helping us to obtain accurate and trustworthy results in our meta-analysis.

### 2.8. Statistical Analysis

In this NMA, a random-effects model was applied to account for the diversity of music therapy types included [41]. The analysis was performed using the frequentist approach in MetaInsight (version 5.1.0, National Institute for Health Research Complex Reviews Support Unit, London, UK), a web-based NMA tool that utilizes the *netmeta* package in R for statistical calculations [42]. Initially, forest plots and network diagrams were created to display all pairwise comparisons among the included studies. This was followed by the generation of forest plots summarizing the standardized mean differences in depression improvement and dropout rate risks among elderly individuals with dementia. These plots compared the differences between each type of music therapy and the control groups [43]. The effects were represented in the form of point estimates and 95% confidence intervals (95% CI) [43]. The types of music therapy were ranked based on their efficacy, with both direct and indirect comparison results presented in tabular form. Inconsistencies within the data were evaluated using specific tests, and statistical significance was defined as a two-sided *p*-value of less than 0.05.

### 2.9. Sensitivity Analysis Approach

To ensure the robustness of our findings, we undertook two separate sensitivity analyses. The initial analysis involved excluding each study, in turn, to determine if any individual study disproportionately affected the overall results. This procedure entailed the sequential omission of each study, followed by an examination to see if such exclusions had any significant impact on the overall study conclusions and the ranking of the interventions. In the second sensitivity analysis, we focused on the correlation coefficient used for the before-and-after depression measurements. Our study initially assumed a correlation coefficient of 0.8, in line with recommendations from the Cochrane Handbook [37]. Recognizing that scholars might use different coefficients, commonly ranging from 0.5 to 0.8 [44], we performed an additional sensitivity analysis. This analysis recalculated the effect sizes of depression changes using a coefficient of 0.5 [44], allowing us to assess the impact of this variable on the outcome’s direction, magnitude, statistical significance, and the ranking of the interventions.

### 2.10. Publication Bias

We checked for possible publication bias following the methods in the Cochrane Handbook for Systematic Reviews of Interventions [19]. A funnel plot was made using Comprehensive Meta-Analysis software, version 4 (Biostat, Englewood, NJ, USA), focusing on comparisons with the control group. Also, we performed an Egger’s regression test to see if there was noticeable publication bias.

## 3. Results

### 3.1. Research Identification and Network Model Construction

**Figure 1:**
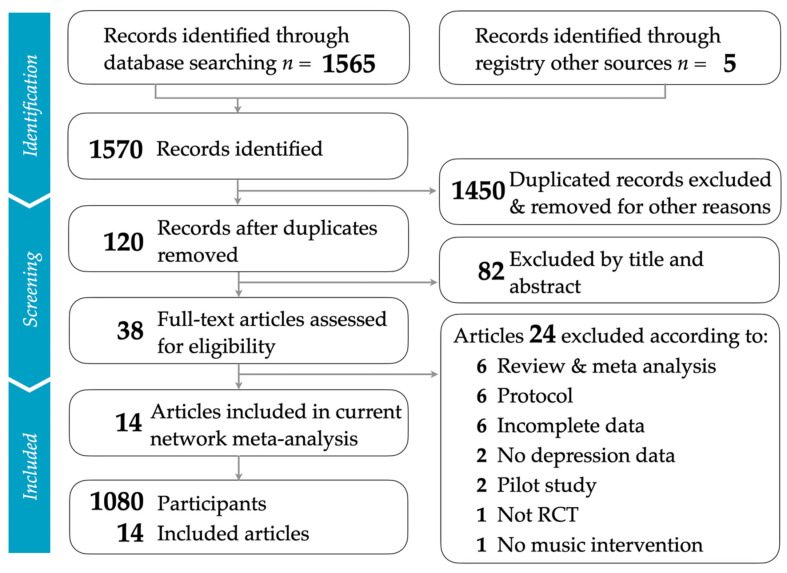
Flowchart illustrating the study selection process, aligned with PRISMA guidelines.

Our study meticulously followed the PRISMA (Preferred Reporting Items for Systematic Reviews and Meta-Analyses) process, as depicted in Figure 1. For more details, refer to the PRISMA NMA (Network Meta-Analysis) checklist in Supplementary Table S1. The number of articles retrieved from various databases can be found in Supplementary Table S2. After eliminating duplicate studies and filtering out irrelevant ones based on their titles and abstracts, we included 14 randomized controlled trials [1,9,12,13,14,45,46,47,48,49,50,51,52,53]. The articles omitted during the final phase, along with the justifications for their exclusion, are detailed in Table S3.

**Figure 2:**
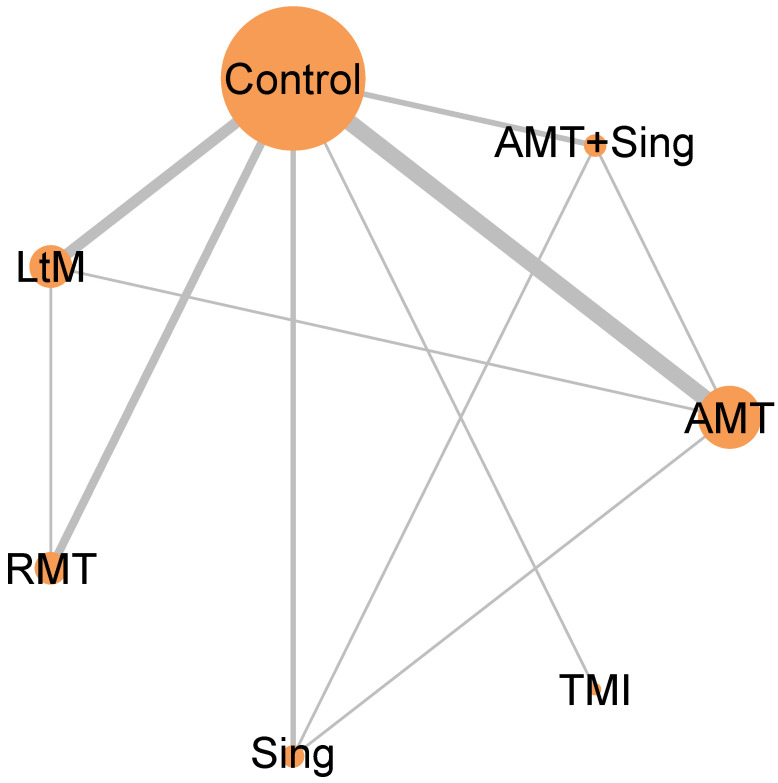
Network diagram illustrating the impact of various music interventions on improving post-activity depression in dementia. The size of each node and the thickness of each line indicate the quantity of trials incorporated in our study. Abbreviations: AMT: Active Music Therapy; RMT: Rhythmic Music Therapy; LtM: Listening to Music; TMI: Tailored Music Intervention.

Our analysis encompassed a total of 14 randomized controlled trials involving 1080 individuals. Based on the included studies, music interventions were categorized into Active Music Therapy (AMT), Sing, Listening to Music (LtM), Rhythmic Music Therapy (RMT), Tailored Music Intervention (TMI), and a combined approach of AMT + Sing. The network model for these music interventions is presented in Figure 2.

The general characteristics of the studies offer a thorough overview, covering a range of aspects from the included research. It details the authors, year of publication for each study, and country of origin. The study design is elaborated, providing insights into the methodologies used. A key focus is on the intervention and control groups, where aspects such as participant count, average age, dementia severity level, and specifics of the music therapy (including session style, types, genres, music titles, and equipment used) are documented. For the control group, the type and descriptions of control measures are provided. Additionally, the section delves into the frequency of treatment, including the duration and period of the intervention, the frequency and length of each session, and the total hours of intervention. The outcomes measured in each study are also summarized. For a more detailed breakdown of these characteristics, please refer to Table 1.

### 3.2. Methodological Quality of the Included Studies

In evaluating the methodological quality of the included studies, we made the following observations across 14 studies: randomization process: 92.9% (13/14) were rated as low risk and 7.1% (1/14) as having some risk. Intervention Adherence: 50% (7/14) were rated as low risk, and 50% (7/14) as having some risk. Missing outcome data: 78.6% (11/14) were assessed as low risk and 21.4% (3/14) as having some risk. Outcome measurement: 92.9% (13/14) were rated as low risk and 7.1% (1/14) as having some risk. Selective reporting: 78.6% (11/14) were rated as low risk, and 21.4% (3/14) as having some risk. Overall risk of bias: 42.9% (6/14) were rated as low risk and 57.1% (8/14) as having some risk (refer to Figure S1). These results demonstrate that while areas like the randomization process and outcome measurement mostly showed a lower risk of bias, over half of the studies presented some level of risk in terms of intervention adherence and overall risk of bias. The details of the risk of bias assessment are provided in Table S4.

### 3.3. Primary Outcome: Active Music Therapy with Singing Most Effective

**Figure 3:**
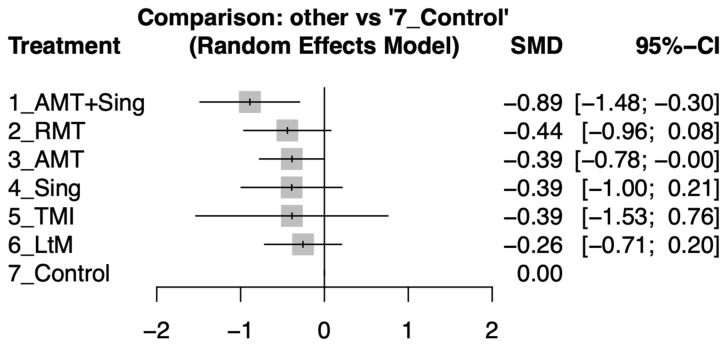
Forest plots illustrating the standardized mean difference (SMD) in depression improvement between different music therapy interventions and control groups in elderly individuals with dementia after the intervention period.

The network meta-analysis results indicated that AMT + Sing was the most effective treatment in improving depressive symptoms in elderly individuals with dementia, showing a significant effect size of −0.89 (95% CI: −1.48 to −0.30). RMT and AMT also showed improvements but were not as significant, with effect sizes of −0.44 (95% CI: −0.96 to 0.08) and −0.39 (95% CI: −0.78 to 0.00), respectively. Simple singing activities (Sing) and TMI each reported an effect size of −0.39 but with wide confidence intervals suggesting less certainty in the results. LtM showed the least impact with an effect size of −0.26 (95% CI: −0.71 to 0.20). The control group serves as the baseline for comparison, with other interventions measured against it (Figure 3). For an in-depth look at the pairwise comparisons between study arms, as detailed in individual studies (see Figure S2).

The music therapy interventions were ranked based on their effect sizes on depression improvement, with AMT + Sing being the most effective, followed by RMT, AMT, Sing, TMI, and LtM in that order. For an in-depth comparison and ranking of the music therapy interventions, kindly refer to Table 2.

### 3.4. Secondary Outcome: Comparable Dropout Rates across Studies

**Figure 4:**
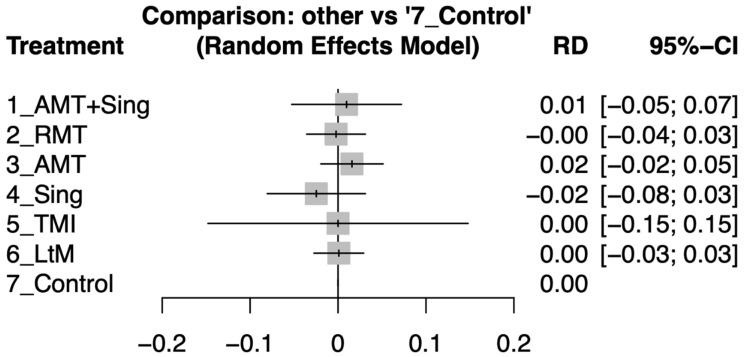
Forest plots depicting the risk difference (RD) in dropout rates between different music therapy interventions and control groups for patients with dementia after the intervention period.

After the intervention period, there was no significant difference in dropout rates between the various music types and the control group, with all risk differences and their 95% confidence intervals overlapping with zero (see Figure 4). For a detailed analysis of the pairwise comparisons between study arms as reported in individual studies (refer to Figure S3).

### 3.5. Inconsistency Test

In our examination of inconsistency tests for depressive symptoms in dementia patients, we observed a *p*-value of 0.04 in the comparison between AMT + Sing to Sing, as shown in Table S5. This marginal statistical significance suggests potential inconsistency in the effectiveness of this specific intervention in alleviating depressive symptoms compared to other interventions. For the assessment of inconsistency in dropout rates, all comparisons yielded results where the 95% CIs included zero, indicating no evidence of inconsistency as documented in Table S6. Given the borderline statistical significance observed between AMT + Sing to Sing, these results should be interpreted with caution. We have conducted sensitivity analyses to ascertain the consistency of these findings, with the results detailed in 3.6—Sensitivity Analyses. This approach aligns with the standards of reporting inconsistency in NMA [19], ensuring a thorough and cautious interpretation of the results.

### 3.6. Sensitivity Analyses

Upon performing the one-study removal sensitivity analysis, the data reinforced the statistical significance of active music therapy combined with singing (AMT + Sing) in the improvement of depressive symptoms among individuals with dementia. The rankings and the clinical relevance of the various music therapy interventions showed consistent patterns, with AMT + Sing consistently emerging as the intervention with notable advantages. For a comprehensive view of these analyses (refer to Figure S4a–n).

In our alternative sensitivity analysis, we recalibrated the pre–post correlation coefficient from 0.8 to 0.5, leading to a revised network comparison (see Figure S5). This adjustment revealed that the effect sizes’ direction, the ranking of interventions, and the overall interpretation of results aligned with those derived using the original coefficient of 0.8 (refer to Figure 3). These analyses collectively affirm that our study’s outcomes are robust, unaffected by either the inclusion or exclusion of specific studies or by variations in assumed values during the analysis process.

### 3.7. Publication Bias

For the funnel plot, refer to Figure S6. The Egger’s test resulted in a *p*-value of 0.144, suggesting an absence of significant publication bias.

## 4. Discussion

### 4.1. Main Findings and Clinical Implications

Our NMA offers a novel perspective on the treatment of depression in dementia, differentiating from traditional meta-analysis by evaluating multiple music therapy approaches simultaneously. This method allows for a comparison of the relative effectiveness of various interventions, which is not possible with standard meta-analyses that typically compare two treatments at a time. The distinct advantage of NMA is highlighted by our findings, which show a spectrum of efficacy across music therapy methods quantified by the SMD. Specifically, AMT + Sing is identified as the most effective approach (SMD = −0.89, 95% CI: −1.48 to −0.30), indicating that the synergistic effect of singing and active engagement in music therapy offers superior benefits for depressive symptoms in dementia compared to other interventions. While effect sizes vary, with AMT + Sing having the most substantial impact and LtM the least, NMA demonstrates that all assessed forms of music therapy, including LtM, have a positive effect, though the magnitude of benefit differs.

Our study leverages NMA to contrast various music interventions, concluding that AMT + Sing is the most effective for reducing depression in dementia; apart from LtM, which has a smaller effect size, other music interventions exhibit moderate efficacy. This is the inaugural study in the literature to address, compare, and rank the effectiveness of different music interventions for this condition.

Furthermore, our analysis found no significant differences in dropout rates across the music interventions compared to control groups. This comprehensive assessment provided by NMA is invaluable for clinicians and caregivers in formulating tailored music therapy prescriptions, underlining the versatility and potential of music therapy to enhance the quality of life for patients with dementia-related depression. The insights gained from this NMA underscore the potential for music therapy to be a key component of a multifaceted therapeutic strategy, aligning with the complexity of care required for dementia.

### 4.2. Interpretation and Contextualization of Results Relative to Existing Research

Our study provides a detailed interpretation and contextualization in the context of existing research. The literature thus far has offered limited insights into the application of music therapy for alleviating dementia-related depression. The meta-analysis by Wang et al., published in 2023 in ‘Complementary Therapies in Clinical Practice’ [54], consolidated data from 21 randomized controlled trials involving 1777 elderly patients with depression up to July 2023. These interventions, lasting six months, included both AMT and PMT programs. Their findings indicated that PMT significantly alleviates depression in comparison to standard care, while the impact of AMT was not substantial. Similarly, the meta-analysis by Zhang et al., released in 2023 in ‘Aging & Mental Health’ [17], analyzed 19 studies, including 16 focusing on depression, encompassing 517 dementia patients. This study did not categorize music interventions by type but reported a potential reduction in depression and anxiety symptoms in dementia patients through music interventions. While Wang et al. provided a broad analysis of AMT and PMT programs without differentiating between dementia and non-dementia depression, Zhang et al. concentrated specifically on dementia. Still, they did not segregate the interventions by type. Both meta-analyses exhibit a positive trend and lay the groundwork for more detailed exploration into which music therapy modalities are most effective, thus highlighting the novelty and necessity of our current research.

Previous studies have frequently underscored the benefits of LtM in alleviating depression in dementia patients [50,51,53]. Our research builds upon this premise by comparing and ranking the impact of various music interventions on depressive symptoms in dementia. Essentially, our analysis seeks not only to reaffirm the effectiveness of LtM in dementia but also to evaluate the relative impact of different music interventions on depression.

### 4.3. Possible Interpretations of Observations

Music therapy is pivotal in dementia care, offering cognitive stimulation, social engagement, and alleviation of depressive symptoms. Our study reveals that the combination of AMT + Sing is the most effective intervention for reducing depressive symptoms in dementia patients. This efficacy likely stems from the enhanced social interaction that singing encourages [49,55]. Studies have shown that engaging in singing and playing musical instruments triggers the release of several neurochemicals, including endorphins and dopamine, which are instrumental in regulating mood and enhancing feelings of pleasure by activating the reward system [56,57,58]. Moreover, singing can also reduce stress hormones such as cortisol [59,60], and singing, recognized for its participatory ease and accessibility, particularly in groups, leads to better quality of life and emotional well-being [9,61]. Singing, integrated with AMT, promotes active patient engagement, which is essential for improving psychological health [49].

Our NMA shows that both RMT and AMT are moderately effective in reducing depressive symptoms in dementia patients (RMT, SMD = −0.44; AMT, SMD = −0.39). RMT uses rhythm-guided movements to provide an enjoyable, relaxed approach to well-being by combining music with physical exercise in a low-stress environment for cognitive engagement [46]. AMT, focusing on therapist-patient interactions, involves patients in creative musical exercises that are multisensory and cognitively stimulating. Using various instruments, AMT activates different brain regions, thus enhancing cognitive functions and promoting neural plasticity [9,45,49].

Moreover, our NMA suggests that LtM has a mild effect on improving depressive symptoms in dementia patients (SMD = −0.26), corroborating research that highlights the stress-reducing properties of music interventions [62,63]. Soothing melodies, such as classical music or nature sounds, help create a peaceful environment, facilitating mental and physical relaxation and offering physiological benefits like reduced heart rate and blood pressure [47,63].

Additionally, Our NMA indicates that dropout rates for music therapy interventions are similar to standard care, likely due to their engaging and user-friendly nature [64]. This ease of participation is essential in dementia care to keep participants engaged. Music therapy sessions, typically led by professional therapists, are both therapeutic and socially engaging, enhancing their acceptability [13,52]. RMT, for example, combines movement with music, providing enjoyable exercise-like benefits and a sense of accomplishment, which helps maintain participant motivation [9]. These findings suggest that music therapy is a practical and well-tolerated option in dementia care, offering a holistic approach that enhances overall therapeutic outcomes.

In summary, our research underscores the value of integrating socially engaging music therapies, such as AMT + Sing, into dementia care. By combining various therapeutic modalities, we more effectively address the complex facets of dementia-related depression. This integrated approach has the potential not only to improve depressive symptoms but also to enhance cognitive function and life quality, making it an integral component of comprehensive dementia care.

### 4.4. Limitations

Our NMA uncovers the prospective benefits of music therapy in reducing depressive symptoms in dementia patients. Nevertheless, several limitations must be acknowledged in interpreting our findings. One primary challenge is the variability in standardization and guidelines across the included studies, complicating the process of consistent comparison and synthesis. Moreover, the duration of music interventions varied widely among the studies, and long-term follow-up research is needed. The inclusion of patients from diverse populations may also introduce variations in dementia characteristics and unique age differences among the studies, further complicating the analysis. Another concern is the higher dropout rates observed in the elderly population, which could potentially skew the results. To assure the reliability of our findings, we conducted thorough examinations of the fourteen studies incorporated into our analysis. Our consistency checks and sensitivity analyses did not identify any single study or group of studies as a source of inconsistency or instability in the results. This suggests that our conclusions are robust and reliable at the current evidence level, without undue influence from any individual study. Despite these limitations, the implications of our study for the daily care and psychological well-being of dementia patients are significant. Future research should focus on developing standardized treatment approaches and conducting long-term follow-up studies to evaluate the impact of music interventions more comprehensively on depressive symptoms in dementia.

## 5. Conclusions

The findings of our study suggest that various music therapy interventions effectively reduce depressive symptoms in dementia patients. Notably, the combined approach of AMT + Sing appears to have the most robust effect, which we attribute to the potential for enhanced social interaction. These results offer new perspectives for dementia care, suggesting a promising direction for further research and clinical application.

## Figures and Tables

**Table 1:** Summarizes the effectiveness of music interventions in alleviating depression in dementia, including details of the trials conducted.

								Intervention Group		Control Group				Frequency of Treatment			
Authors and Year	Country	Study Design	Comparison	*n*	*Dropouts*	Age, Mean (SD)	Dementia Severity	Session Style	Type of Music	Control Type	Control Descriptions	Outcomes	Duration of Intervention	Period (Weeks)	Frequency (Times/Week)	Duration (Hours)	Total Hours
Baker et al., 2022 [1]	Australia	Multi-RCT	GMT	45	32/77	86.5 (7.20)	Severe	AMT Sing AMT + Sing	Patients Preferences	Active	Group game	MADRS	45 min/twice a week/13 weeks (pre) 45 min/once a week/13 weeks (post)	≥12	≥2	≥24	29.25
			RCS	62	20/82												
			GMT + RCS	57	22/79												
			Control	50	30/80												
Biasutti et al., 2021 [12]	Italy	RCT	Music	20	5/25	83.95 (7.84)	Mild	AMT	Improvisation	Active	Gymnistic activities	GDS	70 min/twice a week/6 weeks	<12	≥2	<24	14
			Control	25	1/26	85.12 (6.14)											
Ceccato et al., 2012 [45]	Italy	RCT	Music	27	0/27	85.50 (5.90)	Moderate	AMT	Special compositions	Waitlist	Standard Care and Waitlist	GDS	45 min/twice a week/24 weeks	≥12	≥2	≥24	36
			Control	23	0/23	87.20 (7.10)											
Cheung et al., 2018 [46]	Hong Kong	Multi-RCT	MM	45	13/58	85.71 (6.68)	Moderate	RMT LtM	Multiple music	Passive	Social activity	GDS	40 min/twice a week/6 weeks	<12	≥2	<24	8
			LtM	40	14/54	84.50 (6.82)											
			Control	39	14/53	85.58 (7.46)											
Cheung et al., 2022 [13]	Hong Kong	Cluster-RCT	Music	55	0/55	79.53 (8.53)	Moderate	RMT	Patients Preferences	Waitlist	Waitlist	CSDD	30–45 min/3 times a week/12 weeks	≥12	≥2	≥24	27
			Control	45	0/45												
Chu et al., 2014 [9]	Taiwan	RCT	Music	49	3/52	82.00 (6.80)	Moderate	AMT + Sing	Improvisation	Passive	Usual nursing home care	CSDD	30 min/twice a week/6 weeks	<12	≥2	<24	6
			Control	51	1/52												
Delphin-Combe et al., 2013 [47]	France	RCT	Music	12	0/12	79.20 (6.90)	Moderate	TMI	Multiple music	Passive	Board games	MADRS	30 min/5 times a week/2 weeks	<12	≥2	<24	5
			Control	12	0/12	79.00 (6.70)											
Giovagnoli et al., 2017 [48]	Italy	Multi-RCT	Music	13	4/17	73.92 (7.74)	Moderate	AMT	Improvisation	Active	Cognitive training	BDI	45 min/twice a week/12 weeks	≥12	≥2	<24	18
			Control	13	4/17	73.50 (5.96)											
Giovagnoli et al., 2018 [49]	Italy	Multi-RCT	Music	23	0/23	74.30 (5.70)	Moderate	AMT	Improvisation	Active	Standard care	NPI	45 min/twice a week/24 weeks	≥12	≥2	≥24	36
			Control	22	0/22	72.00 (7.30)											
Guétin et al., 2009 [50]	France	RCT	Music	14	1/15	85.20 (6.00)	Mild	LtM	Multiple music	Passive	Rest and reading	GDS	20 min/once a week/24 weeks	≥12	<2	<24	8
			Control	12	3/15	86.90 (5.20)											
Liu et al., 2021 [14]	Taiwan	RCT	Music	25	0/25	86.60 (4.50)	Mild	RMT	Old songs	Passive	Rest and reading	GDS	60 min/once a week/12 weeks	≥12	<2	<24	12
			Control	25	0/25	86.90 (5.70)											
Pérez-Ros et al., 2019 [51]	Spain	RCT	Music	47	0/47	80.06 (7.63)	Moderate	LtM	Patients Preferences	Passive	Standard care	CSDD	60 min/5 times a week/8 weeks	<12	≥2	≥24	40
			Control	72	0/72	80.80 (7.36)											
Pongan et al., 2017 [52]	France	RCT	Music	31	0/31	78.80 (7.43)	Mild	Sing	Patients Preferences	Passive	Painting	GDS	120 min/once a week/12 weeks	≥12	<2	≥24	24
			Control	28	0/28	80.20 (5.71)											
Raglio et al., 2015 [53]	Italy	Multi-RCT	MT	31	0/31	81.70 (7.80)	Moderate	LtM AMT	Improvisation Patients Preferences	Passive	Standard care	CSDD	30 min/twice a week/10 weeks	<12	≥2	<24	10
			LtM	32	0/32	81.00 (7.60)											
			Control	35	0/35	82.40 (6.80)											

Abbreviations: RCT: Randomized Controlled Trial; N: Number; MT: Music Therapy; MM: Music Movement; SA: Social Activity; GMT: Group Music Therapy; RCS: Recreational Choral Singing; RMT: Rhythmic Music Therapy; AMT: Active Music Therapy; LtM: Listening to Music; TMI: Tailored Music Intervention; SPK: Speaker; NPI: Neuropsychiatric Inventory; GDS: Geriatric Depression Scale; CSDD: Cornell Scale for Depression in Dementia; MADRS: Montgomery–Åsberg Depression Rating Scale; BDI: Beck Depression Inventory.

**Table 2:** Pairwise comparison and ranking of various music Interventions in reducing depression among individuals with dementia.

AMT + Sing						
−0.45 [−1.24; 0.34]	RMT					
−0.50 [−1.15; 0.15]	−0.05 [−0.69; 0.59]	AMT				
−0.50 [−1.24; 0.24]	−0.05 [−0.85; 0.74]	0.00 [−0.66; 0.66]	Sing			
−0.50 [−1.79; 0.79]	−0.05 [−1.31; 1.20]	−0.00 [−1.21; 1.21]	−0.00 [−1.30; 1.29]	TMI		
−0.63 [−1.38; 0.11]	−0.19 [−0.81; 0.44]	−0.13 [−0.70; 0.43]	−0.14 [−0.89; 0.62]	−0.13 [−1.37; 1.10]	LtM	
−0.89 [−1.48; −0.30]	−0.44 [−0.96; 0.08]	−0.39 [−0.78; 0.00]	−0.39 [−1.00; 0.21]	−0.39 [−1.53; 0.76]	−0.26 [−0.71; 0.20]	Control

Table 2 presents the remission rates associated with music therapy interventions that were studied. The larger the negative value, the more effective the intervention was at reducing depressive symptoms. Effect Size represented by SMD and 95% CIs. Abbreviations: AMT: Active Music Therapy; RMT: Rhythmic Music Therapy; LtM: Listening to Music; TMI: Tailored Music Intervention; Color ■: Intervention; ■: Control; ■: Effect Size.

## Data Availability

The data relevant to this study can be found within the article and its Supplementary Files. Regarding conflicts of interest, the authors affirm that there are none.

## Supplementary Materials

The following supporting information is available for download at: https://www.mdpi.com/article/10.3390/ejihpe14020024/s1, specifically tailored for our study on depression in dementia and the intervention of music therapy: Table S1: PRISMA checklist for network meta-analysis; Table S2: Keywords and search strategy results across various databases; Table S3: Studies excluded from the analysis along with the reasons for their exclusion; Table S4: Comprehensive quality assessment of studies included in the analysis; Table S5: Results of inconsistency tests for the improvement of depressive symptoms in dementia patients through music therapy; Table S6: Results of inconsistency tests for the risk difference in dropout rates among dementia patients receiving music therapy; Figure S1: Summary of the quality assessment for included studies; Figure S2: The forest plot of pairwise comparisons for the improvement of depressive symptoms in dementia patients through music therapy; Figure S3: The forest plot of pairwise comparisons for the risk difference of dropout rates in music therapy studies; Figure S4: Sensitivity analysis employing the one-study removal method; Figure S5: Sensitivity analysis adjusting the pre-post correlation coefficient from 0.8 to 0.5; Figure S6: Funnel plot of all paired comparisons involving the common comparator, the control group.
