# Supplementary material for: Multifaceted Music Therapy for Depression in Dementia: A Network Meta-Analysis of Randomized Controlled Trials
Source: Eur J Investig Health Psychol Educ. 2024 Feb 11;14(2):351–67. doi: 10.3390/ejihpe14020024 (PMC10887713; doi:10.3390/ejihpe14020024)
Supplement: Supplementary file 1 [file ejihpe-14-00024-s001.zip › ejihpe-2827786-supplementary.pdf]

Table S1 - PRISMA for network meta-analysis checklist

Section and Topic	#	Checklist item	Location
**Title**			
Title	1	Identify the report as a systematic review incorporating a network meta-analysis (or related form of meta-analysis).	Title
**Abstract**			
Structured summary	2	Provide a structured summary including, as applicable: Background: main objectives / Methods: data sources; study eligibility criteria, participants, and interventions; study appraisal; and synthesis methods, such as network meta-analysis. / Results: number of studies and participants identified; summary estimates with corresponding confidence/credible intervals; treatment rankings may also be discussed. Authors may choose to summarize pairwise comparisons against a chosen treatment included in their analyses for brevity. / Discussion/conclusions: limitations; conclusions and implications of findings. / Other: primary source of funding; systematic review registration number with registry name.	Abstract
**Introduction**			
Rationale	3	Describe the rationale for the review in the context of what is already known, including mention of why a network meta-analysis has been conducted.	Introduction / 1st and 2nd paragraph
Objectives	4	Provide an explicit statement of questions being addressed, with reference to participants, interventions, comparisons, outcomes, and study design (PICOS).	Introduction / 3rd paragraph
**Methods**			
Protocol & registration	5	Indicate whether a review protocol exists and where it can be accessed; and, if available, provide registration information, including registration number.	Materials and Methods/ 1st paragrph
Eligibility criteria	6	Specify study characteristics (e.g., PICOS, length of follow-up) and report characteristics (e.g., years considered, language, publication status) used as criteria for eligibility, giving rationale. Clearly describe eligible treatments included in the treatment network, and note whether any have been clustered or merged into the same node (with justification).	Materials and Methods/ Inculusion and exculusion
Information sources	7	Describe all information sources (e.g., databases with dates of coverage, contact with study authors) in the search and date last searched.	Table S2
Search	8	Present full electronic search strategy for at least one database, including any limits used, such that it could be repeated.	Table S2
Study selection	9	State the process for selecting studies (i.e., screening, eligibility, included in systematic review, and, if applicable, included in the meta-analysis).	Materials and Methods / Study Identification / Inculusion and exculusion criteria
Data collection	10	Describe method of data extraction from reports (e.g., piloted forms, independently, in duplicate) and any processes for obtaining and confirming data.	Materials and Methods / Data extraction

Data items	11	List and define all variables for which data were sought (e.g., PICOS, funding sources) and any assumptions and simplifications made.	Materials and Methods / Data extraction and conversion
Network geometry	S1	Describe methods used to explore the geometry of the treatment network under study and potential biases related to it. This should include how the evidence base has been graphically summarized for presentation, and what characteristics were compiled and used to describe the evidence base to readers.	Materials and Methods / Modeling for network meta-analysis
Risk of bias within	12	Describe methods used for assessing risk of bias of individual studies (including specification of whether this was done at the study or outcome level), and how this information is to be used in any data synthesis.	Materials and Methods / Quality appraisal
Summary measures	13	State the principal summary measures (e.g., risk ratio, difference in means). Also describe the use of additional summary measures assessed, such as treatment rankings, as well as modified approaches used to present summary findings from meta-analyses.	Materials and Methods / Outcome
Planned methods of analysis	14	Describe the methods of handling data and combining results of studies for each network meta-analysis. This should include, but not be limited to: Handling of multi-arm trials; Selection of variance structure; Selection of prior distributions in Bayesian analyses; and Assessment of model fit.	Materials and Methods / Statistical analyses
Assessment of inconsistency	S2	Describe the statistical methods used to evaluate the agreement of direct and indirect evidence in the treatment network(s) studied. Describe efforts taken to address its presence when found.	Materials and Methods / Statistical analyses
Risk of bias across	15	Specify any assessment of risk of bias that may affect the cumulative evidence.	Materials and Methods / Publication bias
Additional analyses	16	Describe methods of additional analyses if done, indicating which were pre-specified. This may include, but not be limited to, the following: Sensitivity or subgroup analyses; Meta-regression analyses; Alternative formulations of the treatment network; and Use of alternative prior distributions for Bayesian analyses (if applicable).	Materials and Methods / Sensitivity analyses
**Results**			
Study selection	17	Give numbers of studies screened, assessed for eligibility, and included in the review, with reasons for exclusions at each stage, ideally with a flow diagram.	Results / Study identification Figure 1, Table S2, Table S3
Network structure	S3	Provide a network graph of the included studies to enable visualization of the geometry of the treatment network.	Fugre 2
Network geometry	S4	Provide a brief overview of characteristics of the treatment network. This may include commentary on the abundance of trials and randomized patients for the different interventions and pairwise comparisons in the network, gaps of evidence in the treatment network, and potential biases reflected by the network structure.	Results / Network model formation / Figure 2
Study characteristics	18	For each study, present characteristics for which data were extracted (e.g., study size, PICOS, follow-up period) and provide the citations.	Table 1
Risk of bias within	19	Present data on risk of bias of each study and, if available, any outcome level assessment.	Table S4, Figure S1, Methodological quality

Results of individual studies	20	For all outcomes considered (benefits or harms), present, for each study: (1) simple summary data for each intervention group, and (2) effect estimates and confidence intervals. Modified approaches may be needed to deal with information from larger networks.	Table 1
Synthesis of results	21	Present results of each meta-analysis done, including confidence/credible intervals. In larger networks, authors may focus on comparisons versus a particular comparator (e.g. placebo or standard care), with full findings presented in an appendix. League tables and forest plots may be considered to summarize pairwise comparisons. If additional summary measures were explored (such as treatment rankings), these should also be presented.	Outcomes / Figure 3, Figure 4, Figure S2, Figure S3, Table 2
Exploration for inconsistency	S5	Describe results from investigations of inconsistency. This may include such information as measures of model fit to compare consistency and inconsistency models, P values from statistical tests, or summary of inconsistency estimates from different parts of the treatment network.	Inconsistency test<br>Table S5, Table S6
Risk of bias across	22	Present results of any assessment of risk of bias across studies for the evidence base being studied.	Publicaton bias, Figure S6
Additional analyses	23	Give results of additional analyses, if done (e.g., sensitivity or subgroup analyses, meta-regression analyses, alternative network geometries studied, alternative choice of prior distributions for Bayesian analyses, and so forth).	Sensitivity analysis / Figure S4, Figre S5
**Discussion**			
Summary of evidence	24	Summarize the main findings, including the strength of evidence for each main outcome; consider their relevance to key groups.	Discussion<br>Findings and implications
Limitations	25	Discuss limitations at study and outcome level (e.g., risk of bias), and at review level (e.g., incomplete retrieval of identified research, reporting bias). Comment on the validity of the assumptions, such as transitivity and consistency. Comment on any concerns regarding network geometry (e.g., avoidance of certain comparisons).	Discussion<br>Linitations
Conclusions	26	Provide a general interpretation of the results in the context of other evidence, and implications for future research.	Conclusion
**Funding**			
Funding	27	Describe sources of funding for the systematic review and other support (e.g., supply of data); role of funders for the systematic review. This should also include information regarding whether funding has been received from manufacturers of treatments in the network and/or whether some of the authors are content experts with professional conflicts of interest that could affect use of treatments in the network.	Funding

Table S2 - Keywords and search results in different databases ***1570***

Database	Keyword	Date	Results
PubMed	music AND depression AND dementia	2023.10.11	206
Embase	music AND depression AND dementia	2023.10.11	460
Cochrane CENTRAL	music AND depression AND dementia	2023.10.11	138
Web of Science	music AND depression AND dementia	2023.10.11	279
			1083

Database	Keyword	Date	Results
PubMed	music AND depression AND alzheimer	2023.10.11	91
Embase	music AND depression AND alzheimer	2023.10.11	224
Cochrane CENTRAL	music AND depression AND alzheimer	2023.10.11	80
Web of Science	music AND depression AND alzheimer	2023.10.11	92
			487

Database	Keyword	Date	Results
PubMed	music AND depression AND dementia AND ('random' OR 'randomized' OR 'randomised')	2023.10.11	73
Embase	music AND depression AND dementia AND ('random' OR 'randomized' OR 'randomised')	2023.10.11	162
Cochrane CENTRAL	music AND depression AND dementia AND ('random' OR 'randomized' OR 'randomised')	2023.10.11	129
Web of Science	music AND depression AND dementia AND ('random' OR 'randomized' OR 'randomised')	2023.10.11	114
			478

Database	Keyword	Date	Results
PubMed	music AND depression AND alzheimer AND ('random' OR 'randomized' OR 'randomised')	2023.10.11	35
Embase	music AND depression AND alzheimer AND ('random' OR 'randomized' OR 'randomised')	2023.10.11	74
Cochrane CENTRAL	music AND depression AND alzheimer AND ('random' OR 'randomized' OR 'randomised')	2023.10.11	71
Web of Science	music AND depression AND alzheimer AND ('random' OR 'randomized' OR 'randomised')	2023.10.11	34
			214

Table S3 Excluded studies and reasons

No.	First Author / Publication Year	Title	Journal/Book	Exclusion reasons
1	Bakerjian et al., 2020	The Impact of Music and Memory on Resident Level Outcomes in California Nursing Homes	Journal of the American Medical Directors Association	Not RCT
2	Buard et al., 2021	Randomized controlled trial of neurologic music therapy in Parkinson's disease: research rehabilitation protocols for mechanistic and clinical investigations	Trials	Protocol
3	Clark et al., 2020	"It's Feasible to Write a Song": A Feasibility Study Examining Group Therapeutic Songwriting for People Living With Dementia and Their Family Caregivers	Frontiers in Psychology	Incomplete data
4	Davison et al., 2016	A personalized multimedia device to treat agitated behavior and improve mood in people with dementia: A pilot study	Geriatr Nurs	Pilot study
5	Gaviola et al., 2020	Impact of individualised music listening intervention on persons with dementia: A systematic review of randomised controlled trials	Australasian journal on ageing	Review & meta analysis
6	Jung et al., 2023	Effect of internet-based vs. in-person multimodal interventions on patients with mild to moderate Alzheimer's disease: a randomized, cross-over, open-label trial	Frontiers in Public Health	No music intervention
7	Kwak et al., 2020	Findings From a Prospective Randomized Controlled Trial of an Individualized Music Listening Program for Persons With Dementia	Journal of Applied Gerontology	Incomplete data
8	Lai et al., 2016	Interdisciplinary collaboration in the use of a music-with-movement intervention to promote the wellbeing of people with dementia and their families: Development of an evidence-based intervention protocol	Nursing & Health Sciences	Protocol
9	Loi et al., 2022	Music and Psychology & Social Connections Program: Protocol for a Novel Intervention for Dyads Affected by Younger-Onset Dementia	Brain Sciences	Protocol
10	Mahendran et al., 2017	Art therapy and music reminiscence activity in the prevention of cognitive decline: Study protocol for a randomized controlled trial	Trials	Protocol
11	McCreedy et al., 2022	Pragmatic Trial of Personalized Music for Agitation and Antipsychotic Use in Nursing Home Residents With Dementia	Journal of the American Medical Directors Association	Incomplete data
12	Na et al., 2019	A systematic review and meta-analysis of nonpharmacological interventions for moderate to severe dementia	Psychiatry Investigation	Review & meta analysis
13	Noone et al., 2019	Meta-analysis of psychosocial interventions for people with dementia and anxiety or depression	Aging & mental health	Review & meta analysis
14	Raglio et al., 2008	Efficacy of music therapy in the treatment of behavioral and psychiatric symptoms of dementia	Alzheimer Disease & Associated Disorders	Incomplete data
15	Raglio et al., 2015	Effects of music and music therapy on mood in neurological patients	World Journal of Psychiatry	Review & meta analysis
16	Sánchez et al., 2016	Comparing the Effects of Multisensory Stimulation and Individualized Music Sessions on Elderly People with Severe Dementia: A Randomized Controlled Trial	Journal of Alzheimer's Disease	Incomplete data
17	Särkämö et al., 2014	Music perception and cognition: development, neural basis, and rehabilitative use of music	Wiley Interdisciplinary Reviews: Cognitive Science	Incomplete data

No.	First Author / Publication Year	Title	Journal/Book	Exclusion reasons
18	Scott S. et al., 2016	A scoping review of music and anxiety, depression and agitation in older people with dementia in residential facilities and specialist care units	European Geriatric Medicine	Review & meta analysis
19	Svansdottir et al., 2006	Music therapy in moderate and severe dementia of Alzheimer's type: a case-control study	Int Psychogeriatr	No depression data
20	Tan et al., 2018	Study protocol for a randomized controlled trial of choral singing intervention to prevent cognitive decline in at-risk older adults living in the community	Frontiers in Aging Neuroscience	Protocol
21	Tang et al., 2013	The effectiveness of nursing management on improving health outcomes for hospitalized older adults with delirium: A systematic review protocol	JBI Database of Systematic Reviews and Implementation Reports	Protocol
22	Thornley et al., 2016	Music therapy in patients with dementia and behavioral disturbance on an inpatient psychiatry unit: results from a pilot randomized controlled study	International Psychogeriatrics	Pilot study
23	Valdiglesias et al., 2017	Is Salivary Chromogranin A a Valid Psychological Stress Biomarker During Sensory Stimulation in People with Advanced Dementia?	Journal of Alzheimer's Disease	No depression data
24	Zhang et al., 2023	Does music intervention relieve depression or anxiety in people living with dementia? A systematic review and meta-analysis	Aging & Mental Health	Review & meta analysis

Table S4 - Detailed quality assessment of included studies using Cochrane risk of bias 2 tool

First author & Year	Randomization process	Intervention adherence	Missing outcome data	Outcome measurement	Selective reporting	Overall RoB
Baker et al., 2022	L	S[2]	S	L	L	S
Biasutti et al., 2021	L	S[2]	S	L	S	S
Ceccato et al., 2012	L	S[3]	L	L	S	S
Cheung et al., 2018	L	L[2]	L	L	S	L
Cheung et al., 2022	S	L[2]	S	L	S	S
Chu et al., 2014	L	L[2]	S	L	L	S
Delphin-Combe et al., 2013	L	S[2]	S	L	S	S
Giovagnoli et al., 2017	L	S[2]	S	L	L	L
Giovagnoli et al., 2018	L	S[2]	S	L	S	S
Guétin et al., 2009	L	S[2]	S	L	L	L
Liu et al., 2021	L	S[2]	L	L	L	L
Pérez-Ros et al., 2019	L	S[2]	L	L	L	S
Pongan et al., 2017	S	S[2]	S	L	S	S
Raglio et al., 2015	L	S[2]	S	L	L	S

1 The study employed a waitlist control group design, which resulted in a more balanced comparison among different groups.

2 The differences in protocols among various groups may affect adherence and outcome.

3 Both groups were randomized to receive exercise interventions, and the study design utilized a balanced protocol, which minimized the impact on adherence.

H, high risk of bias; L, low risk of bias; S, some risk of bias.

Table S5. Results of inconsistency tests for the standardized mean difference in depression improvement among dementia patients receiving music therapy

Comparison	Studies	NMA	Direct	Indirect	Difference	95CI-L	95CI-U	p-value
AMT:AMT+Sing	1	0.50	0.39	0.62	-0.23	-1.54	1.08	0.73
AMT:Control	6	-0.39	-0.41	-0.11	-0.31	-1.73	1.11	0.67
AMT:LtM	1	-0.13	-0.25	-0.07	-0.17	-1.35	1.01	0.78
AMT:RMT	0	0.05	-	0.05	-	-	-	-
AMT:Sing	1	0.00	0.41	-0.47	0.89	-0.44	2.21	0.19
AMT:TMI	0	0.00	-	0.00	-	-	-	-
AMT+Sing:Control	2	-0.89	-1.07	0.17	-1.24	-2.92	0.44	0.15
AMT+Sing:LtM	0	-0.63	-	-0.63	-	-	-	-
AMT+Sing:RMT	0	-0.45	-	-0.45	-	-	-	-
AMT+Sing:Sing	1	-0.50	0.02	-1.66	1.68	0.08	3.28	0.04
AMT+Sing:TMI	0	-0.50	-	-0.50	-	-	-	-
LtM:Control	4	-0.26	-0.23	-0.43	0.19	-1.29	1.67	0.80
RMT:Control	3	-0.44	-0.44	-0.49	0.05	-1.93	2.03	0.96
Sing:Control	2	-0.39	-0.29	-0.94	0.65	-1.01	2.31	0.44
TMI:Control	1	-0.39	-0.39	-	-	-	-	-
LtM:RMT	1	0.19	0.14	0.23	-0.10	-1.35	1.16	0.88
LtM:Sing	0	0.14	-	0.14	-	-	-	-
LtM:TMI	0	0.13	-	0.13	-	-	-	-
RMT:Sing	0	-0.05	-	-0.05	-	-	-	-
RMT:TMI	0	-0.05	-	-0.05	-	-	-	-
Sing:TMI	0	0.00	-	0.00	-	-	-	-

95CI-L: lower limit of 95% confidence interval; 95CI-U: upper limit of 95% confidence interval; NMA: network meta-analysis.

Table S6. Inconsistency test results for the risk difference in dropout rates when applying music therapy to alleviate depression in dementia patients.

Comparison	Studies	NMA	Direct	Indirect	Difference	95CI-L	95CI-U	p-value
AMT:AMT+Sing	1	0.01	0.14	-0.03	0.17	0.00	0.34	0.05
AMT:Control	6	0.02	0.01	0.07	-0.07	-0.19	0.06	0.30
AMT:LtM	1	0.01	0.00	0.03	-0.03	-0.11	0.05	0.50
AMT:RMT	0	0.02	-	0.02	-	-	-	-
AMT:Sing	1	0.04	0.17	0.01	0.16	0.00	0.33	0.05
AMT:TMI	0	0.02	-	0.02	-	-	-	-
AMT+Sing:Control	2	0.01	0.01	0.00	0.01	-0.20	0.22	0.91
AMT+Sing:LtM	0	0.01	-	0.01	-	-	-	-
AMT+Sing:RMT	0	0.01	-	0.01	-	-	-	-
AMT+Sing:Sing	1	0.03	0.03	0.03	0.00	-0.17	0.17	1.00
AMT+Sing:TMI	0	0.01	-	0.01	-	-	-	-
LtM:Control	4	0.00	0.00	0.07	-0.07	-0.21	0.07	0.32
RMT:Control	3	0.00	0.00	-0.03	0.03	-0.24	0.29	0.83
Sing:Control	2	-0.02	-0.02	-0.05	0.03	-0.17	0.23	0.79
TMI:Control	1	0.00	0.00	-	-	-	-	-
LtM:RMT	1	0.00	0.04	0.00	0.03	-0.13	0.20	0.68
LtM:Sing	0	0.03	-	0.03	-	-	-	-
LtM:TMI	0	0.00	-	0.00	-	-	-	-
RMT:Sing	0	0.02	-	0.02	-	-	-	-
RMT:TMI	0	0.00	-	0.00	-	-	-	-
Sing:TMI	0	-0.02	-	-0.02	-	-	-	-

95CI-L: lower limit of 95% confidence interval; 95CI-U: upper limit of 95% confidence interval; NMA: network meta-analysis.

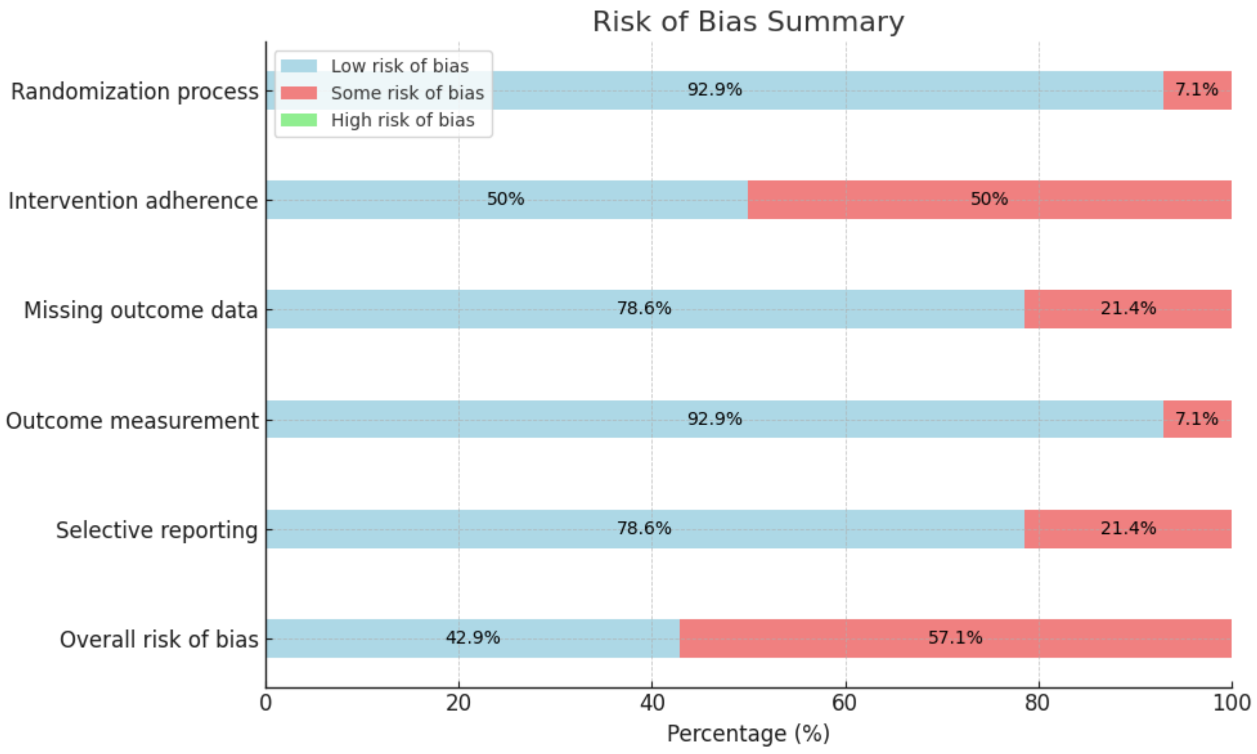

Figure S1. Summary of quality assessment of studies included in the network meta-analysis using Cochrane risk of bias 2 tool

Fig S2. Individual study results (for all studies) grouped by treatment comparison

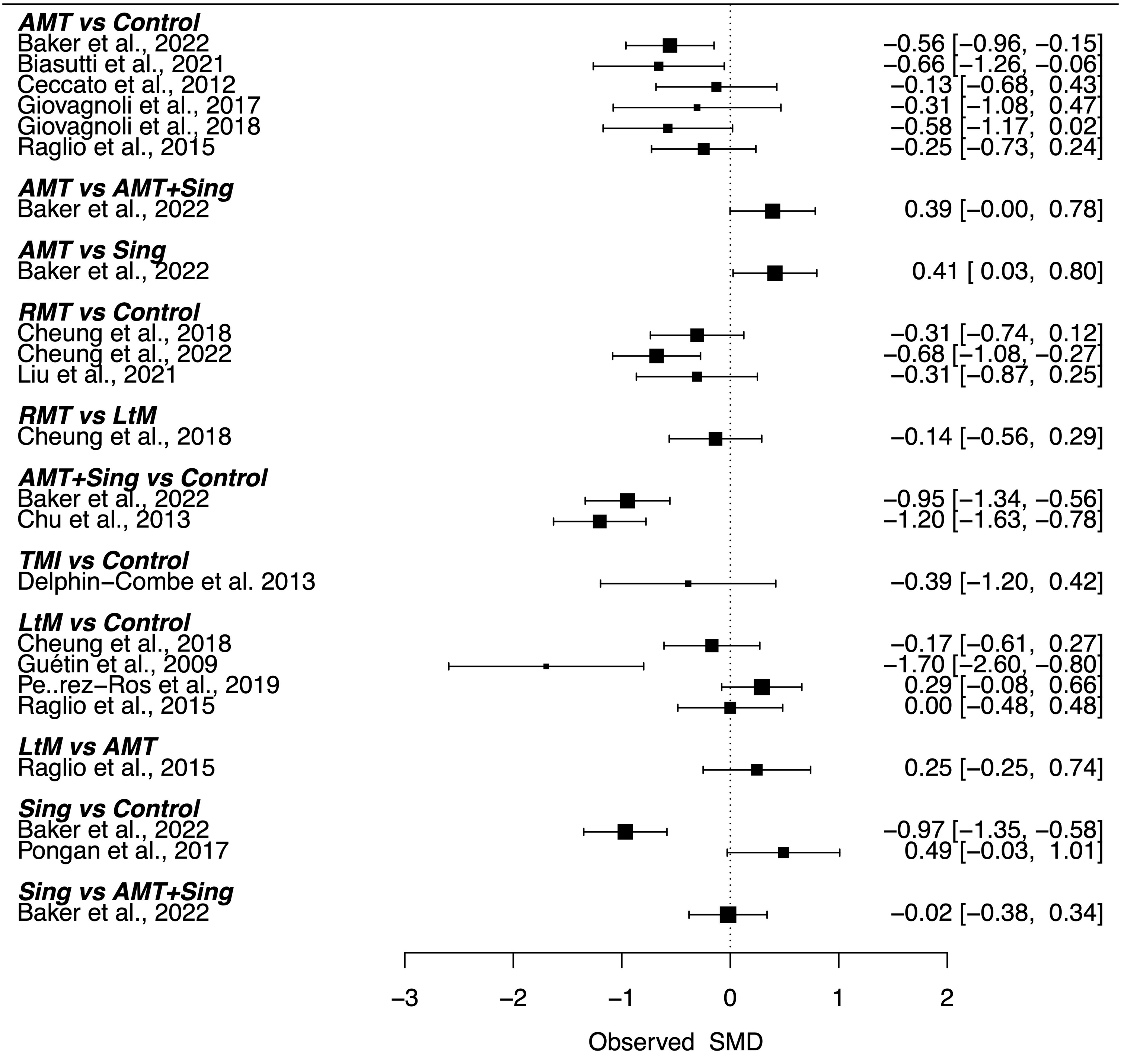

Fig S2. The forest plot of pairwise comparisons for different music interventions aimed at alleviating depression in individuals with dementia, as derived from the included trials, showcases the standardized mean difference (SMD) in improvement of depression symptoms.

Fig S3. **Individual study results (for all studies) grouped by treatment comparison**

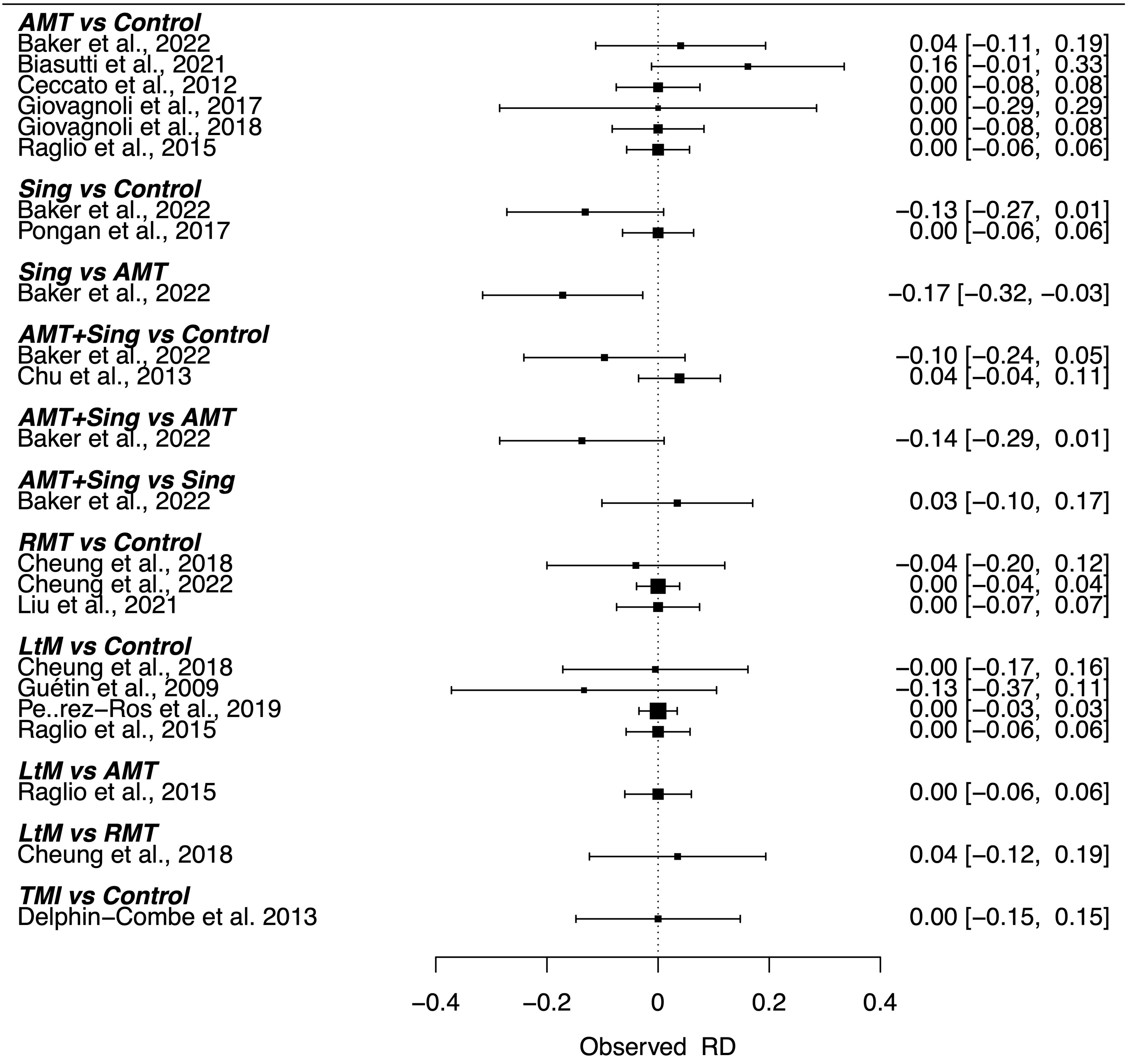

Fig S3. The forest plot of pairwise comparisons for different music interventions in patients with dementia and depression, retrieved from the included trials, demonstrates the risk difference (RD) of dropout rates from the experiments. None of the comparisons reached statistical significance.

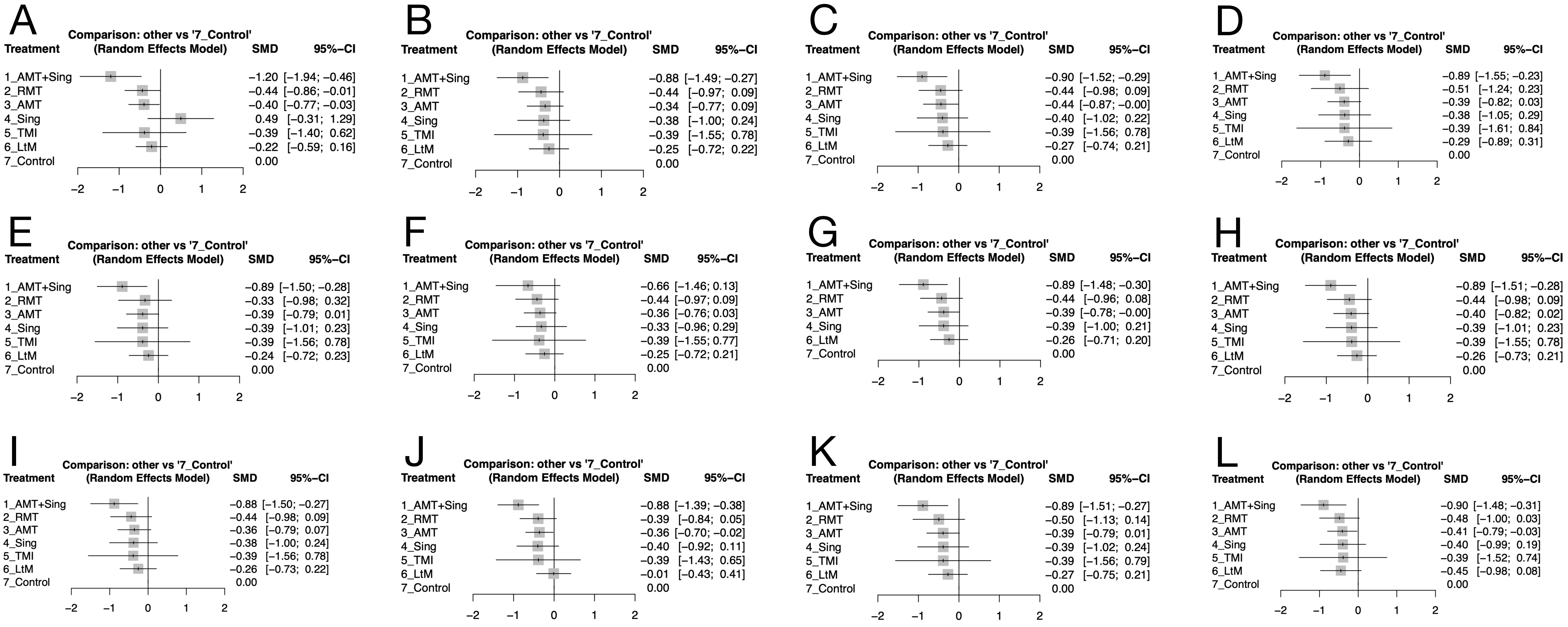

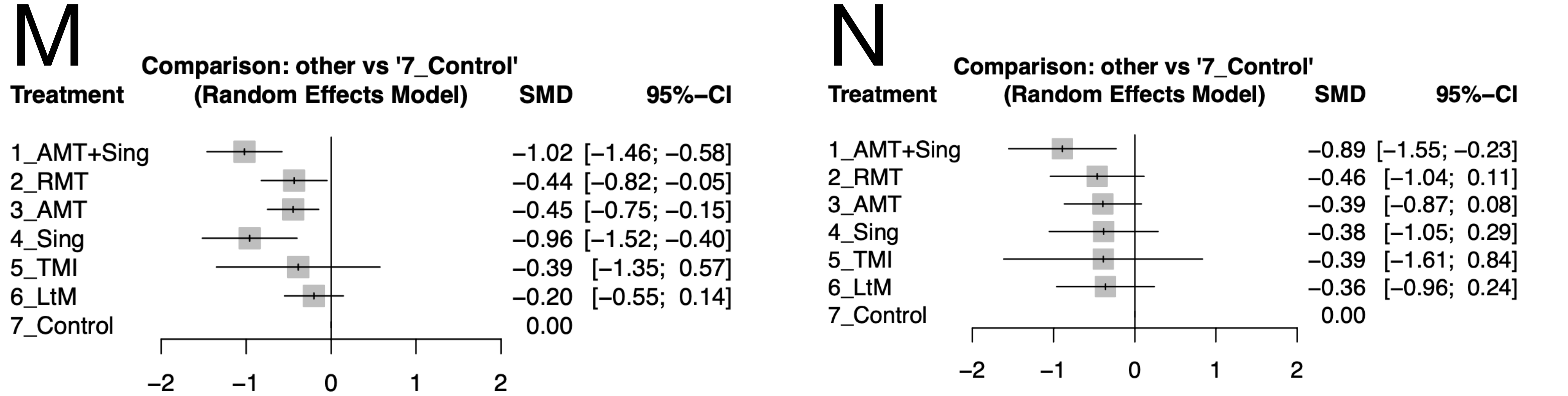

Studies Excluded One by One (Referenced Alphabetically)

A. Baker et al., 2022
B. Biasutti et al., 2021
C. Ceccato et al., 2012
D. Cheung et al., 2018
E. Cheung et al., 2022
F. Chu et al., 2013
G. Delphin-Combe et al. 2013
H. Giovagnoli et al., 2017
I. Giovagnoli et al., 2018
J. Guétin et al., 2009
K. Liu et al., 2021
L. Pérez-Ros et al., 2019
M. Pongan et al., 2017
N. Raglio et al., 2015

Fig-S4 displays forest plots for sensitivity analysis using the one-study removal method, where each of the 14 studies included was sequentially omitted according to their publication year (labeled a to n).

Fig-S4

Figure S5

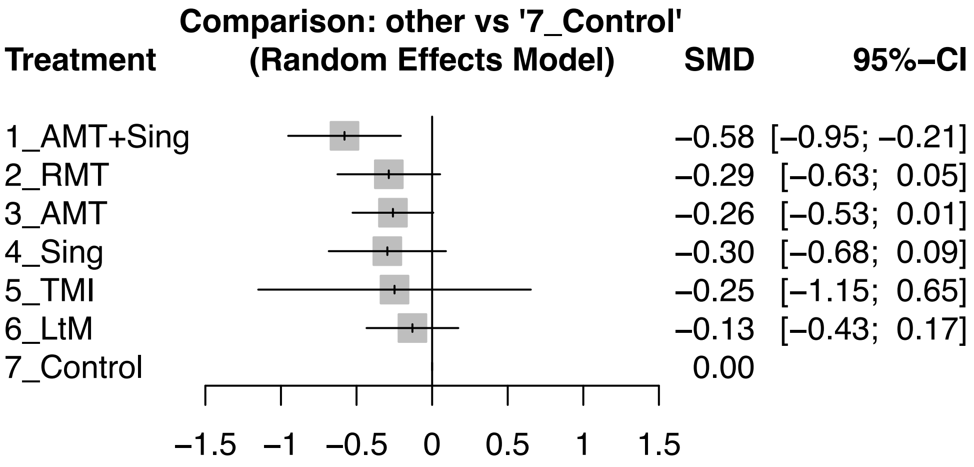

Fig. S5 - Forest plot displaying the improvement in depression symptoms in dementia patients after receiving music therapy interventions, presented as standardized mean differences (SMDs). The pre-post correlation coefficient used in the calculation of data was changed from 0.8 used in Figure 3 to 0.5 in this figure as a sensitivity analysis. The ranking and clinical interpretations remained unchanged compared to Figure 3. This suggests that the conclusions of our study remain unchanged despite different assumptions regarding the coefficient used for transformation.

Figure S6

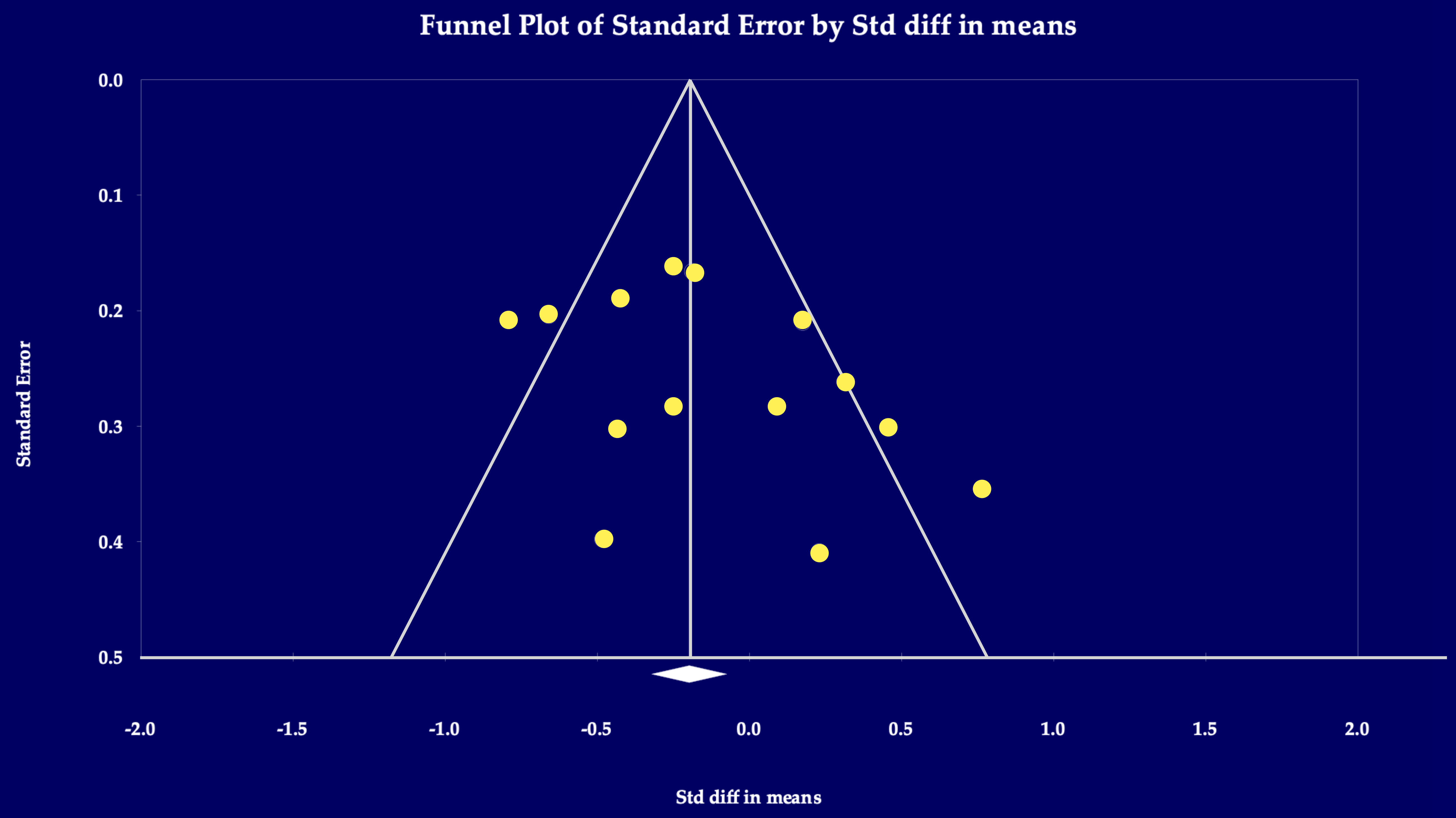

FigS6. Egger's test revealed no significant publication bias (intercept = 2.39, 95% CI (-0.94 to 5.72), t = 1.56, df = 12, p = 0.072 [1-tailed], p = 0.144 [2-tailed]), indicating that the results are reliable.
